# Social Perception of Natural Background Radiation and Its Implications for Public Health Communication

**DOI:** 10.3390/healthcare14101424

**Published:** 2026-05-21

**Authors:** Juliánna Szakács, Mihai Ioan Georgescu, Gellért-Gedeon Deák, Eszter Bajkó, Simona Toncean Florentina, Florina Ruta, Călin Avram

**Affiliations:** 1 Department of Biophysics, Faculty of Medicine, George Emil Palade University of Medicine, Pharmacy, Science, and Technology of Targu Mures, Gheorghe Marinescu Street No. 38, 540142 Targu Mures, Romania; julianna.szakacs@umfst.ro; 2 Department of Obstetrics and Gynecology, Faculty of Medicine and Pharmacy, “Dunărea de Jos” University of Galați, 800008 Galati, Romania; 3 Department of Obstetrics Gynecology, Braila City Emergency Hospital, 810325 Braila, Romania; 4 Faculty of Medicine, George Emil Palade University of Medicine, Pharmacy, Science, and Technology of Targu Mures, Gheorghe Marinescu Street No. 38, 540142 Targu Mures, Romania; gellertgedeon@gmail.com (G.-G.D.); bajkoeszter4@gmail.com (E.B.); 5 Department of Medical Radiology and Imaging, Târgu Mureș County Emergency Clinical Hospital, 540136 Targu Mures, Romania; 6 Department of Management, Dr Eugen Nicoara Municipal Hospital, 545300 Reghin, Romania; s.boariu@yahoo.com; 7 Department of Community Nutrition and Food Safety, George Emil Palade University of Medicine, Pharmacy, Science, and Technology of Targu Mures, Gheorghe Marinescu Street No 38, 540142 Targu Mures, Romania; florina.ruta@umfst.ro; 8 Department of Medical Informatics and Biostatistics, George Emil Palade University of Medicine, Pharmacy, Science, and Technology of Targu Mures, Gheorghe Marinescu Street No. 38, 540142 Targu Mures, Romania

**Keywords:** environmental radiation, risk perception, health communication, institutional trust, public attitudes, radiation protection information

## Abstract

**Highlights:**

**What are the main findings?**

- Public perception of natural background radiation is moderate to high despite only moderate levels of knowledge.
- Higher perceived risk is primarily associated with low institutional trust and a lack of professional medical information.

**What are the implications of the main findings?**

- Radiation risk communication should provide clear and differentiated explanations of natural versus medical radiation while strengthening trust in healthcare institutions.
- Active involvement of healthcare professionals is essential to manage uncertainty and prevent unjustified avoidance behaviors.

**Abstract:**

Background: Public perception of environmental (natural background) radiation represents an important challenge for public health communication, as risk perception is often influenced more by information quality and institutional trust than by objective exposure levels. Methods: A cross-sectional survey was conducted among 481 respondents using a structured questionnaire assessing self-perceived knowledge, information sources, perceived radiation risk, institutional trust, and health-related attitudes. Results: Significant gender differences were observed in self-reported knowledge about radioactivity, with men more frequently reporting higher knowledge levels than women (*p* < 0.001), while no significant differences emerged between urban and rural respondents; logistic regression analysis showed that lower perceived risk was associated with lack of medical information (OR = 0.32, 95% CI: 0.14–0.71) and absence of avoidance behavior (OR = 0.23, 95% CI: 0.11–0.47), whereas low trust in medical institutions was associated with higher odds of perceiving natural background radiation as dangerous (OR = 1.84, 95% CI: 1.21–2.80). Conclusions: Effective radiation risk communication requires more than the dissemination of information; it must also address public concerns, enhance institutional trust, and provide clear, credible, and accessible health-related messages. Tailored communication strategies are essential to bridge the gap between expert knowledge and public perception.

## 1. Introduction

The issue of environmental radiation occupies a special place in the risk culture of modern societies. Natural background radiation, including terrestrial, cosmic, and radon-related exposure, is a permanent component of human life, yet for the general public it often remains an invisible, abstract, and emotionally charged phenomenon. For this reason, international public health and radiation protection bodies increasingly treat radiation communication not only as a technical matter, but also as a core prevention and health education task [1,2,3,4,5,6].

Natural background radiation refers to ionizing radiation of natural origin to which the population is naturally, permanently, and continuously exposed. The population’s exposure to natural background radiation occurs both externally and internally [1,2,3,4].

External exposure is caused primarily by cosmic radiation from outer space and terrestrial radiation emitted by natural radionuclides present in the Earth’s crust [3].

Cosmic radiation originates in outer space and reaches the Earth’s surface after particles of cosmic origin interact with the atmosphere. Exposure to cosmic radiation depends primarily on altitude and, to a lesser extent, on latitude. People living at high altitudes in mountainous areas or who travel frequently by plane are exposed to a higher dose of cosmic radiation [5,6].

Terrestrial radiation is emitted by natural radionuclides present in the Earth’s crust. The most significant of these are uranium, thorium, and potassium—specifically uranium-238, thorium-232, and potassium-40—which are found in soil, rocks, and, sometimes, in building materials. Through their natural decay, these radionuclides emit ionizing radiation, contributing to human external exposure [5,6].

Natural background radiation depends on the geological composition of the region, as certain types of rocks and soils contain higher concentrations of natural radionuclides [6]. A very important component of natural background radiation is radon (an alpha emitter), a naturally occurring, colorless, and odorless radioactive gas that results primarily from the decay of uranium-238 found in soil and rocks. Radon can enter buildings through cracks in the foundation, walls, floors, or plumbing and can accumulate in poorly ventilated spaces. The World Health Organization (WHO) considers radon to be one of the most significant natural sources of exposure to ionizing radiation and a risk factor for lung cancer, particularly in the case of prolonged exposure to high concentrations indoors [1].

Although radiation can be quantified through dosimetric and epidemiological methods, public responses are rarely shaped by exposure data alone. Radiation is commonly associated with uncertainty, lack of control, delayed health effects, and catastrophic imaginaries, therefore risk judgments are often influenced by affective and symbolic factors in addition to scientific evidence. Classic and contemporary research alike shows that public assessments of radiological risk frequently differ from expert judgment, and this discrepancy has major consequences for trust, communication, and behavioural responses [7,8,9].

Recent studies focusing on radon and natural indoor exposure confirm that risk perception is strongly influenced by message framing, institutional credibility, and the social context in which information is received. Research from Slovenia demonstrated that societal attitudes toward indoor radon are closely linked to protective intentions, while experimental work also showed that different formulations of the same radon risk message can significantly alter perceived seriousness and willingness to act [10,11]. In parallel, studies on domestic radon mitigation and occupational exposure highlight that barriers to action are not merely informational, but also practical, psychosocial, and organizational in nature [12,13,14,15,16,17].

The communication environment further complicates public understanding. People encounter information about radiation through news stories, social media, interpersonal narratives, medical encounters, and institutional announcements, which often provide fragmented or unevenly contextualized explanations. As a result, individuals may conflate natural background radiation, medical imaging, occupational exposure, and nuclear emergencies into a single undifferentiated threat category. Studies on media narratives and radiation communication therefore emphasize that effective public messages must be credible, understandable, and adapted to the audience’s prior beliefs and concerns [18].

Beliefs about radiation can influence health behaviour, willingness to undergo di-agnostic imaging, acceptance of environmental measurements, and trust in healthcare and regulatory institutions. Examining how the participant interprets environmental radiation is therefore important not only from a theoretical perspective, but also for prevention, health literacy, and public policy. Against this background, the present questionnaire-based study aimed to explore the participants perceptions of environmental radiation, attitudes related to physical and psychological effects, and communication needs, while also taking into account features of the living environment, the presence of natural and artificial radiation sources, and broader public health concerns related to cumulative exposure [19,20,21,22,23,24].

## 2. Materials and Methods

The study employed a cross-sectional observational design based on self-reported data collected through an anonymous online questionnaire. Data collection was carried out between October and December 2025. Participants were recruited using a non-probabilistic convenience sampling approach, by distributing the questionnaire via social media platforms and academic communication channels, which may introduce self-selection and reporting bias. Participation was voluntary, and no financial incentives were provided.

The inclusion criterion was complete questionnaire completion. Out of a total of 484 respondents, three participants were excluded due to incomplete self-reported data, resulting in a final sample of 481 participants. Incomplete responses were excluded using a complete-case analysis approach.

The study was conducted in accordance with the Declaration of Helsinki and was approved by the Ethics Committee of the University of Medicine, Pharmacy, Science and Technology “George Emil Palade” of Târgu Mureș (UMPHST) on 17 June 2025, approval number 3819. All participants were informed about the study objectives and methodology, and informed consent was obtained prior to accessing the questionnaire. No personally identifiable data were collected, and all data were securely stored.

The questionnaire, administered via Google Forms, consisted of 40 items organized into sections covering sociodemographic characteristics, knowledge and awareness of radiation effects, perceptions and attitudes toward radiation, as well as health status and additional radiation exposure. The questionnaire included single-choice items, multiple-choice items, and Likert-scale items designed to assess respondents’ knowledge, attitudes, and perceptions. The questionnaire was developed based on a review of the relevant literature and was preliminarily evaluated through a pilot study to assess item clarity and comprehensibility.

In the initial phase, the questionnaire was applied to a restricted group of participants to evaluate item clarity and understanding. Based on the feedback obtained during this pilot phase, minor adjustments were made, including the modification of certain groups of variables and the reduction in response options for selected items. Following these revisions, the final version of the questionnaire was used in the present study.

Statistical analyses were performed using GraphPad Prism software, version 10.6.1. (Boston, MA, USA). The normality of data distributions was assessed using the Shapiro–Wilk test. Continuous variables were reported as mean ± standard deviation, while categorical variables were presented as frequencies and percentages. The chi-square test was used to compare proportions, and the Mann–Whitney U test was applied for non-normally distributed continuous variables. The dependent variable in the logistic regression analysis was high perceived risk of natural background radiation (yes/no). Independent variables included socio-demographic characteristics (sex, age, education level, and place of residence), self-perceived knowledge about radioactivity, interest in the health effects of radiation, institutional trust, and selected health-related variables. The results were expressed as odds ratios (ORs) with 95% confidence intervals (95% CI) and corresponding *p*-values. For all analyses, *p*-value ≤ 0.05 was considered statistically significant.

## 3. Results

The sample size is 481 people, 348 women and 133 men. The participants were between 18 and 90 years old. The respondents’ places of residence were divided between urban and rural areas, with a predominance of rural areas. In terms of living environment, 55% of respondents live on side streets, 37% on main roads, 5% near major intersections, and 2% near playgrounds.

A statistically meaningful difference in mean age was observed between female and male respondents, with women showing a slightly higher average age than men (*p* = 0.0389). By contrast, no significant disparities were identified between the two groups with regard to educational attainment or type of residence (urban versus rural) (Table 1).

Respondents living in urban areas more frequently indicated that their homes were situated near secondary or major roads, whereas alternative residential settings were more commonly reported in rural areas. This comparison points to marked differences between urban and rural environments in terms of housing location (*p* < 0.001). Furthermore, dwelling construction materials varied considerably depending on residential context, with rural participants more often reporting houses built from wood or unfinished brick. These variations were statistically significant (*p* < 0.001) (Table 1).

The level of self-reported knowledge regarding radioactivity revealed clear differences between genders, with men more frequently declaring a higher level of knowledge than women (*p* < 0.001). A similar pattern was observed in the perception of statements related to radiation (*p* = 0.050). In contrast, the urban–rural comparative analysis did not indicate any statistically significant differences (*p* = 0.293). Overall, the data suggest a moderate variability in the level of knowledge among participants, without the emergence of systematic differences between the analyzed groups (Table 2).

All participants reported being aware of natural background radiation, regardless of gender or place of residence. This universal familiarity suggests that the concept is broadly known within the study participants and may provide a common foundation for further understanding of radiation-related risks and effects.

Table 3 summarizes differences in perceptions and attitudes toward radiation across gender and place of residence. Sex-based differences were observed for several indicators, including interest in additional medical information, perceived risk of natural background radiation, concerns about exceeding permissible limits, trust in medical institutions, and avoidance behaviors, with women generally reporting higher levels of concern and precaution, while men expressed greater trust and interest in information. Differences related to place of residence were less consistent, reaching significance only for perceived risk levels and concerns about limit exceedance.

Table 4 outlines health-related characteristics and additional exposures among participants. Statistically significant differences between sexes were observed for thyroid disorders (*p* = 0.002), headache frequency over the past three months (*p* < 0.00001), and chronic fatigue symptoms (*p* < 0.00001), all of which were reported more frequently by women than by men.

Table 5 presents the results of the multivariable logistic regression analysis examining factors associated with perceiving natural background radiation as dangerous. Lower odds of high risk perception were observed among respondents reporting a lack of medical information regarding radiation doses and associated risks (OR = 0.32, 95% CI: 0.14–0.71, *p* = 0.005) and among those who did not avoid products perceived as slightly radioactive (OR = 0.23, 95% CI: 0.11–0.47, *p* < 0.0001). In contrast, low trust in medical institutions regarding the monitoring and reduction in radiation exposure was significantly associated with higher odds of perceiving natural background radiation as dangerous (OR = 1.84, 95% CI: 1.21–2.80, *p* = 0.005).

## 4. Discussion

The relationship between self-assessed uncertainty and actual knowledge gaps is supported by international literature. Bastiani et al. conducted a patient survey related to medical imaging and concluded that patients generally have limited knowledge about medical radiation exposure and its risks. The authors concluded that better patient education could have a positive effect on understanding the risks [25]. A similar picture emerges from a review by Ribeiro et al., which found that patients’ knowledge of ionizing radiation associated with medical imaging was inadequate in several studies [26]. Singh and colleagues also found in an Australian radiology setting that patients have little knowledge of radiation doses and associated risks and receive little information from healthcare professionals [27]. These findings indicate that insufficient knowledge about radiation represents a phenomenon consistently observed across different cultural contexts and healthcare systems, suggesting the presence of common mechanisms related to risk communication. Taken together, these findings support the concept of limited radiation literacy despite relatively high awareness of the topic, highlighting a gap between exposure to information and meaningful understanding. It is also important to note that insufficient understanding of radiation risk may reflect not only limitations in risk communication, but also more fundamental gaps in education, including limited early exposure to basic concepts of physics, probability, and risk interpretation.

The tabulated results nuance this picture further. Although almost all respondents reported at least some prior exposure to the topic, self-perceived preparedness remained modest, and a significant gender difference emerged: men rated themselves as better informed than women (*p* = 0.001; Table 2). At the same time, previous active information-seeking was common in both groups, suggesting that the problem is not complete lack of contact with the topic, but rather the limited consolidation of that information into stable and confident understanding. This reinforces the distinction between awareness and functional literacy, where familiarity does not necessarily translate into informed decision-making.

Health literacy related to radiation does not simply mean that an individual has “heard” about radioactivity and radiation exposure, but also that they are able to interpret the differences between various sources of exposure, understand the dose relationships in medical imaging, and weigh the risks and benefits. In this context, radiological literacy can be regarded as a specific component of health literacy, encompassing both conceptual knowledge and decision-making skills. This indicates partial familiarity rather than robust scientific literacy, which is highly relevant when communication targets are designed for the general participants.

Based on our research, it can be said that participants engage in interest-driven but fragmented information gathering, often through unstructured online and social media sources, which does not necessarily lead to stable knowledge. This pattern is consistent with contemporary models of digital information consumption, which are characterized by brief and heterogeneous exposures.

The dominance of the internet, and social media in particular, suggests that much of the knowledge related to radiation comes from unstructured, professionally unregulated channels. This does not necessarily mean that online information is inaccurate, but due to the nature of the digital environment, there is a greater likelihood of loss of context, sensationalist framing, and the spread of partial or misleading information. Consequently, the online environment may amplify risk perceptions even when the initial information is factually correct. Since the concept of radiation is often associated in everyday language with nuclear accidents, cancer, and “invisible dangers”, information consumed in the online space can have a particularly strong emotional impact. Radiation risk perception is shaped not only by factual knowledge but also by uncertainty, perceived lack of control, symbolic threat, and trust in institutions, rather than by objective exposure parameters. This distribution is extremely significant from a science communication perspective. The dominance of digital space indicates that a significant portion of lay knowledge about radiation does not come directly from professionally controlled health communication. The difference between the social interpretation of radiological risks and the expert approach stems in part from the fact that the public and the expert community use different language and risk frameworks [10]. The effective communication is based on understandable, contextualized information that presents the balance of benefits and risks [28,29,30,31].

A lack of communication does not necessarily indicate a lack of trust; rather, it indicates that credible individuals are not present with sufficient intensity or clarity in the flow of information to the public [32,33].

The socio-demographic table also suggests that exposure context may shape communication needs. This finding underscores the importance of incorporating contextual factors into risk communication strategies. Respondents from urban areas more often reported living near secondary roads or main roads, whereas rural respondents more frequently lived in quieter residential settings, and this distribution was statistically significant (*p* < 0.001; Table 1). This matters because everyday environmental cues can influence what people interpret as pollution, contamination, or health risk, thereby affecting how radiation-related information is framed and understood. These contextual differences suggest that risk communication strategies should be adapted to specific environmental and social settings rather than assuming a homogeneous audience.

Risk communication activities often aim to shape risk perception, improve understanding, and strengthen trust; but the effectiveness of communication depends largely on whether the messages are clear, consistent, and relevant to the audience. The communication on radiological topics is particularly difficult and therefore requires a simple, transparent, and well-prepared institutional presence [19,28,29].

One of the most interesting areas of social perception of radiation is the relationship to natural background radiation (Table 3). Fifty-nine percent of respondents consider natural background radiation to be a moderate risk, 32% consider it a high risk, and 9% consider it a low risk. In addition, 190 respondents believe that the level of natural background radiation exceeds the permissible limits. Women also consider natural background radiation to be more dangerous than men in this respect.

These data are noteworthy in several respects. These findings indicate that radio-logical risk perception is influenced more by mental representations than by the physical characteristics of the source. First, they indicate that “natural” characteristics alone do not reduce the perception of risk. In lay thinking, it is not uncommon to assume that anything of natural origin appears less dangerous. Here, however, natural background radiation does not appear in this way; the majority of respondents consider it to be at least moderately dangerous. This suggests that radiation as a concept carries a strong general sense of threat, regardless of whether its source is natural or artificial. This reinforces the idea that perceived danger is shaped more by cognitive and emotional factors than by objective exposure levels.

The subgroup analyses strengthen this conclusion. The observed differences suggest the presence of sociopsychological variations in the way environmental risks are interpreted. Women were significantly more likely than men to rate natural background radiation as highly dangerous, and they were also more likely to believe that surrounding radiation levels exceed acceptable limits. In addition, respondents from rural areas tended to perceive natural background radiation as more dangerous than urban respondents, while urban respondents more often chose the moderate-risk category. These differences suggest that risk communication should not treat the public as homogeneous; instead, messages may need to be calibrated for different experiential and social contexts.

Secondly, belief in background radiation levels exceeding “acceptable limits” may indicate that the concepts of regulation, dose measurement, and risk communication are unclear to respondents.

Thirdly, risk perception is strongly linked to a precautionary attitude. In total, 396 respondents would avoid slightly radioactive products, and 428 respondents would agree to radiation measurements being carried out in their place of residence. This dual pattern clearly shows that the attitude of the participants is not manifested in the form of passive anxiety, but also carries behavioral orientation: some people would avoid products considered to be risky, while supporting the institutional practice of monitoring and measurement. This preventive attitude is not necessarily unfavorable from a health policy perspective, but it can easily turn into excessive risk avoidance if it is not accompanied by appropriate, differentiated professional communication.

Such precautionary behavior may be adaptive but requires balanced communication to prevent disproportionate risk avoidance. It is particularly noteworthy that the “natural” origin does not in itself reduce the sense of danger. The risk perception related to radiation is not simply based on technical parameters, but is organized around uncertainty, lack of control, and the symbolic meaning of threat [7,34,35].

The detailed distribution of symptom frequency is likewise important (Table 4). These results should be interpreted with caution, as the observed relationships do not necessarily imply a direct causal link with radiation exposure. This highlights the need to avoid causal interpretations in a cross-sectional design.

Women not only reported thyroid disease more often, but also more frequent headaches and chronic fatigue, whereas smoking prevalence did not differ significantly between women and men. This pattern argues against simplistic interpretations that would attribute symptom differences to a single behavioural factor and instead supports a broader biopsychosocial reading of symptom reporting in the context of perceived environmental risk.

Fifty-nine participants reported known thyroid disease, headaches appeared in various frequency categories, and chronic fatigue was also reported with significant frequency. Gender differences are significant in all three areas: women report thyroid disease, headaches, and chronic fatigue more frequently.

Thyroid disease, headaches, and chronic fatigue are extremely common, multifactorial phenomena. Their occurrence can be influenced by hormonal, lifestyle, psychological, social, and other health factors.

At the same time, the tables show an important latent demand for professional explanation: a very large majority of participants wanted more information from medical personnel about radiation, with this expectation being even stronger among women than among men. This finding emphasizes the key role of healthcare professionals in improving radiation-related health literacy. This suggests that low refusal rates should not be interpreted as absence of concern; rather, people may still comply with recommended investigations while feeling insufficiently informed about exposure, safety, and benefit–risk balance.

One of the most concrete health behavior consequences of radiation fear could be the refusal of medical imaging tests. Ninety-seven percent of respondents did not refuse medical tests due to radiation fear, and only 3% did so. This suggests that concern does not necessarily translate into avoidance behavior at the participant level.

Based on the results obtained, it appears that there is significant concern and un-certainty regarding radiation, but this does not predominantly translate into strong refusal of treatment. This coexistence of concern and acceptance points to a pragmatic balance in the behavioral responses of the participants. The participants can therefore be both anxious and pragmatic: they may fear radiation, but when it comes to medical examinations, they do not usually refuse them. This is important from a public health perspective, as it suggests that fear can be managed with appropriate information and has not yet led to widespread refusal of necessary diagnostics.

At the same time, a rate of 3% cannot be considered entirely insignificant. If this behavior is real, then for a small but vulnerable group, fear of radiation may be a concrete obstacle to seeking care. It follows that health communication should focus not on reducing panic in general, but on providing targeted information specific to the examination situation: patients should understand how to weigh the diagnostic benefits against the radiation exposure.

However, the smaller proportion of examination refusals cannot be ignored. The communication about radiation risks in healthcare should be based on a clear presentation of the risk-benefit balance, especially in situations where the patient or their relatives are uncertain [36].

Taken together, the findings point to a mixed pattern: the public shows readiness to accept monitoring, interest in receiving more information, and only limited refusal of medical procedures, yet this is accompanied by uncertainty, selective misconceptions, and moderate rather than strong institutional trust. This pattern reflects an intermediate stage in the consolidation of health literacy, in which interest still exceeds the level of established understanding. For this reason, future public health strategies should combine transparent institutional communication with practical educational interventions that explain everyday exposure, natural background radiation, and medical radiation in clearly differentiated terms.

Of the respondents, 156 expressed low confidence, 202 expressed medium confidence, and 123 expressed high confidence that healthcare institutions monitor and reduce radiation exposure. The most common category was medium confidence. This distribution indicates a moderate level of institutional trust, which represents both an opportunity and a vulnerability for public health communication.

The aforementioned communication paradox—namely, that participants receive limited information from healthcare institutions yet expect reliable information from them—is consistent with this finding. It appears that these institutions possess normative legitimacy, while their operational communication remains weak.

Healthcare and policy institutions need to communicate in a more visible, proactive, and accessible way, especially on topics that are technically complex but emotionally charged. Consistent and transparent communication is essential to strengthen trust and improve public understanding.

“Moderate trust” is a vulnerable state from a communication perspective: it pro-vides sufficient legitimacy to rely on institutions as credible actors, but is not strong enough for the public to automatically favor institutional knowledge. In this context, the consistency of messages becomes just as important as their scientific accuracy. The consistent, pre-planned, and easily understandable institutional communication is therefore essential, especially on radiological issues [37,38,39,40].

The level of natural background radiation is not uniform across the globe. It varies depending on altitude, the geological composition of the soil, the type of building materials, the characteristics and ventilation of dwellings, and indoor radon concentrations. For this reason, some regions may have higher levels of natural background radiation without this necessarily indicating artificial contamination or polluting human activity [6].

Internal exposure occurs through the inhalation of air (by inhaling naturally occurring radioactive gases, particularly radon, thoron, and their decay products) and through the ingestion of water and food containing natural radionuclides. The International Atomic Energy Agency (IAEA) notes that radionuclides such as potassium-40, carbon-14, and radium-226 are present in the body, blood, or bones, and that the average natural exposure is approximately 3 mSv/year, with significant variations depending on the geographic region [39,40]. Potassium-40, which occurs naturally in the human body, is an essential element for cell function [5,6,39,40].

Organic foods produced through agriculture and grown in soil containing radio-active minerals may contain small amounts of naturally occurring radioisotopes [40].

Globally, natural background radiation is the main factor contributing to the population’s exposure to ionizing radiation. According to United Nations Scientific Committee on the Effects of Atomic Radiation (UNSCEAR) reports, the average annual dose from natural sources is approximately 2.4 mSv/year, but this value can vary significantly from one region to another, depending on local environmental conditions [5,6]. It is important to distinguish between natural and artificial exposure and to contribute to the assessment of radiological risks to the population and the environment [39].

In addition to natural background radiation, we are also exposed to medical ionizing radiation from both diagnostic procedures and therapeutic interventions [1].

In diagnostic imaging, doses are generally low and are expressed as the effective dose in mSv (milliSieverts), which serves to comparatively estimate the radiation risk associated with different procedures. Conventional X-rays involve low doses, whereas computed tomography (CT) and hybrid PET/CT procedures can result in significantly higher doses. In radiation therapy, the dose is expressed in Grays (Gy), as the goal is to deliver a sufficiently high absorbed dose to the tumor tissue while limiting exposure to healthy tissues. A digital mammogram can expose a patient to approximately 0.28 mSv, a chest X-ray to approximately 0.02–0.1 mSv, and CT scans generally involve higher doses: approximately 1.6 mSv for a head CT, 6.1 mSv for a chest CT, and 7.7 mSv for an abdominal-pelvic CT, approximately 0.005 mSv [39,40].

In conventional external beam radiation therapy, the dose is usually fractionated, meaning it is administered in repeated sessions, typically at 1.8–2.0 Gy per fraction, with total doses that can reach approximately 50–70 Gy in curative treatments, depending on the tumor location, stage, treatment goal, and the tolerance of the organs at risk [40].

The study has certain limitations. The cross-sectional design does not allow for the establishment of causal relationships between the analyzed variables. Data were collected through self-reported measures, which may introduce reporting bias, and the use of an online questionnaire may limit the representativeness of the sample. Additionally, self-assessed knowledge and perception-based responses may introduce subjective bias in the interpretation of the results.

The sample was predominantly composed of female respondents and students, reflecting the recruitment strategy based on academic channels and online platforms. This imbalance may limit the generalizability of the findings to the broader population. Future studies should aim to recruit more diverse and representative samples by employing probabilistic sampling strategies and targeted recruitment of underrepresented groups.

## 5. Conclusions

This study highlights that public perception of natural background radiation is shaped by a combination of general familiarity with the concept of radioactivity and, simultaneously, insufficient knowledge regarding radiation sources, actual exposure levels, and potential health consequences. In public perception, radiation is associated not only with physical exposure but also with factors such as lack of control, uncertainty, perceived invisibility of danger, and possible long-term health effects.

The fact that even a small proportion of respondents avoid medical examinations due to fear of radiation underscores the need for more effective communication in specific clinical contexts, particularly regarding the risk–benefit ratio of medical imaging procedures.

Overall, the findings support the notion that perceptions of natural background radiation are influenced not only by objective information but also by emotional factors, symbolic representations of danger, personal experiences, and institutional trust. Consequently, there is a clear need for structured public communication, using accessible language, that clearly differentiates between natural background radiation, medical exposures, and other radiation sources, thereby enabling informed, balanced, and proportionate decision-making.

## Figures and Tables

**Table 1:** Socio-demographic data of the study participants.

Variables	Women (*n* = 348)	Men (*n* = 133)	*p* Value	Urban (*n* = 321)	Rural (*n* = 160)	*p* Value
Age (mean, SD)	28.42 (13.85)	24.36 (10.66)	0.0389 *	27.98 (13.99)	25.94 (11.24)	0.269 *
Level of education:			0.0872			0.341
Pre-secondary	9 (2.59%)	1 (0.75%)		7 (2.18%)	3 (1.88%)	
Secondary	182 (52.30%)	83 (62.4%)		168 (52.34%)	97 (60.63%)	
Higher education	157 (45.11%)	49 (36.8%)		146 (45.48%)	60 (37.50%)	
Residence			0.388 *	-	-	-
Urban	120 (34.48%)	40 (30.08%)				
Rural	228 (65.52%)	93 (69.92%)				
Where is your residence?			0.3964 *			<0.001 *
Near a major intersection	9 (4.9%)	6 (9.0%)		54 (16.82%)	6 (3.75%)	
Next to the main road	69 (37.7%)	23 (34.3%)		82 (25.55%)	60 (37.50%)	
Next to a playground	4 (2.2%)	0 (0.0%)		8 (2.49%)	0 (0.0%)	
Side street	101 (55.2%)	38 (56.7%)		177 (55.14%)	94 (58.75%)	
What material is it made of?			0.266 *			<0.0001 *
Concrete	132 (37.93%)	61 (45.86%)		142 (44.24%)	51 (31.88%)	
Brick	153 (43.97%)	54 (40.60%)		127 (39.56%)	80 (50.00%)	
Wood	30 (8.62%)	6 (4.51%)		15 (4.67%)	21 (13.13%)	
I don’t know	33 (9.48%)	12 (9.02%)		37 (11.53%)	8 (5.00%)	
Job			0.122 *			0.013 *
Institutional work	47 (13.51%)	11 (8.27%)		45 (12.02%)	13 (8.13%)	
Student	218 (62.64%)	98 (73.68%)		209 (65.11%)	107 (66.88%)	
Office work	38 (10.92%)	12 (9.02%)		33 (10.28%)	17 (10.63%)	
Outdoor physical work	6 (1.72%)	5 (3.76%)		5 (1.56%)	6 (3.75%)	
Indoor physical work	17 (4.89%)	2 (1.50%)		13 (4.05%)	6 (3.75%)	
Work from home	15 (4.31%)	3 (2.26%)		7 (2.18%)	11 (6.88%)	
Retired	7 (2.01%)	2 (1.50%)		9 (2.80%)	0 (0.0%)	

* = Mann–Whitney test.

**Table 2:** Level of knowledge and awareness regarding radiation.

Variables	Women (*n* = 348)	Men (*n* = 133)	*p* Value	Urban (*n* = 321)	Rural (*n* = 160)	*p* Value
How well-informed do you feel about radioactivity?			0.001 *			0.293 *
Not very informed	150 (43.10%)	44 (33.08%)		122 (38.01%)	72 (45.00%)	
Average	140 (40.23%)	46 (34.59%)		131 (40.81%)	55 (34.38%)	
Very	58 (16.67%)	43 (32.33%)		68 (21.18%)	33 (20.63%)	
Have you ever refused a medical examination because you were afraid of radiation?			0.079 *			0.344 *
Yes	13 (3.74%)	4 (3.01%)		11 (3.43%)	3 (1.88%)	
No	248 (71.26%)	108 (81.20%)		245 (76.32%)	117 (73.13%)	
Not applicable	87 (25.00%)	21 (15.79%)		65 (20.25%)	40 (25.00%)	
Have you ever looked into the health effects of radiation?			0.247 *			0.627 *
Yes	275 (79.02%)	112 (84.21%)		256 (79.75%)	131 (81.88%)	
No	73 (20.98%)	21 (15.79%)		65 (20.25%)	29 (18.13%)	
Which of these statements do you think are true?			0.050 *			0.293 *
Food intake results in a measurable—but very small—dose	33 (9.48%)	15 (11.28%)		30 (9.35%)	18 (11.25%)	
Background radiation can be completely eliminated	42 (12.07%)	15 (11.28%)		35 (10.90%)	22 (13.75%)	
Radon may be present in homes	215 (61.78%)	67 (50.38%)		186 (57.94%)	96 (60.00%)	
Air travel can increase the annual dose	58 (16.67%)	36 (27.07%)		70 (21.81%)	24 (15.00%)	

* = Mann–Whitney test.

**Table 3:** Perceptions, attitudes, and opinions regarding radiation.

Variables	Women (*n* = 348)	Men (*n* = 133)	*p* Value	Urban (*n* = 321)	Rural (*n* = 160)	*p* Value
Would you like medical staff to provide you with more information about the radiation doses you’ve been exposed to and the associated risks?			<0.0001			0.621
Yes	317 (91.09%)	103 (77.44%)		277 (86.29%)	143 (89.38%)	
No	20 (5.75%)	10 (7.52%)		22 (6.85%)	8 (5.00%)	
I don’t think so	11 (3.16%)	20 (15.04%)		22 (6.85%)	9 (5.63%)	
How dangerous do you consider natural background radiation to be?			<0.0001			0.023
Low perceived danger	63 (18.10%)	64 (48.12%)		97 (30.22%)	30 (18.75%)	
Moderate perceived danger	159 (45.69%)	40 (30.08%)		130 (40.50%)	68 (42.50%)	
High perceived danger	126 (36.21%)	29 (21.80%)		94 (29.28%)	62 (38.75%)	
Do you think the level of natural background radiation around you exceeds the permissible limits?			<0.0001			0.023
Yes	156 (44.83%)	34 (25.56%)		115 (35.83%)	75 (46.88%)	
No	192 (55.17%)	99 (74.44%)		206 (64.17%)	85 (53.13%)	
Do you think the public should be better informed about radioactivity?			<0.0001			0.999
Yes	333 (95.69%)	112 (84.21%)		297 (92.52%)	148 (92.50%)	
No	15 (4.31%)	21 (15.79%)		24 (7.48%)	12 (7.50%)	
How confident are you that medical institutions monitor and pay attention to reducing radiation exposure?			0.008			0.241
Low confidence	121 (34.77%)	35 (26.32%)		98 (30.53%)	58 (36.25%)	
Moderate confidence	151 (43.39%)	51 (38.35%)		134 (41.74%)	68 (42.50%)	
High confidence	76 (21.84%)	47 (35.34%)		89 (27.73%)	34 (21.25%)	
Would you avoid a product if you found out it was slightly radioactive?			<0.0001			0.311
Yes	300 (86.21%)	96 (72.18%)		260 (81.00%)	136 (85.00%)	
No	48 (13.79%)	37 (27.82%)		61 (19.00%)	24 (15.00%)	
If possible, would you agree to have the background radiation level in your home measured?			0.192			0.167
Yes	314 (90.23%)	114 (85.71%)		281 (87.54%)	147 (91.88%)	
No	34 (9.77%)	19 (14.29%)		40 (12.46%)	13 (8.13%)	

**Table 4:** Health Status and Additional Exposures.

Variables	Women (*n* = 348)	Men (*n* = 133)	*p* Value	Urban (*n* = 321)	Rural (*n* = 160)	*p* Value
Do you have any known thyroid conditions?			0.002			0.258
Yes	54 (15.52%)	5 (3.76%)		40 (12.46%)	19 (11.88%)	
No	247 (70.98%)	110 (82.71%)		232 (72.27%)	125 (78.13%)	
I don’t know	47 (13.51%)	18 (13.53%)		49 (15.26%)	16 (10.00%)	
Have you had frequent headaches in the last 3 months?			<0.0001			0.926
Almost every day	20 (5.75%)	5 (3.76%)		17 (5.30%)	8 (5.00%)	
Monthly	65 (18.68%)	8 (6.02%)		48 (14.95%)	25 (15.63%)	
Never	59 (16.95%)	54 (40.60%)		77 (23.99%)	36 (22.50%)	
Rarely	146 (41.95%)	61 (45.86%)		140 (43.61%)	67 (41.88%)	
Weekly	58 (16.68%)	5 (3.76%)		39 (12.15%)	24 (15.00%)	
Have you experienced symptoms of chronic fatigue?			<0.0001			0.402
Almost every day	48 (13.79%)	9 (6.77%)		35 (10.90%)	22 (13.75%)	
Monthly	63 (18.10%)	14 (10.53%)		46 (14.33%)	31 (19.38%)	
Never	41 (11.78%)	39 (29.32%)		53 (16.51%)	27 (16.88%)	
Rarely	127 (36.49%)	66 (49.62%)		137 (42.68%)	56 (35%)	
Weekly	69 (19.83%)	5 (3.76%)		50 (15.58%)	24 (15%)	
Smoking			0.712			0.306
Yes	125 (35.92%)	44 (33.08%)		120 (37.38%)	49 (30.63%)	
No	223 (64.08%)	89 (66.92%)		201 (62.62%)	111 (69.38%)	
Over the past 5 years, how many times have you traveled by plane (a round-trip journey counts as one flight)?			0.440			0.148
1–5 flights	219 (62.93%)	86 (64.66%)		204 (63.55%)	101 (63.13%)	
6–12 flights	31 (8.91%)	11 (8.27%)		16 (4.98%)	3 (1.88%)	
>12 flights	11 (3.16%)	8 (6.02%)		31 (9.66%)	11 (6.88%)	
Never	87 (25.00%)	28 (21.05%)		70 (21.81%)	45 (28.13%)	

**Table 5:** Multivariable logistic regression analysis of factors associated with high perceived risk of natural background radiation.

Variables	“Is Natural Background Radiation Dangerous?” vs. “Is Natural Background Radiation Not Dangerous?		
	OR	95% CI	*p* Value
Lack of medical information on radiation doses and risks	0.3164	0.1414 to 0.7083	0.0051
No avoidance of slightly radioactive products	0.2317	0.1149 to 0.4673	<0.0001
Low trust in medical institutions regarding radiation exposure reduction	1.8396	1.2068 to 2.8041	0.0046
Perceived lack of public information about radiation	0.3722	0.1220 to 1.1350	0.0823
No daily room ventilation (≥10–15 min)	0.7539	0.4808 to 1.1819	0.2181
Smoking status (current smoker)	0.7992	0.5211 to 1.2256	0.3042

OR, odds ratio; CI, confidence interval.

## Data Availability

The data that support the findings of this study are available from the corresponding author upon reasonable request.
